# Embolization treatment of right pulmonary artery agenisis with patent ductus arteriosus causing pulmonary hypertension and hemoptysis: a case report and literature review

**DOI:** 10.1186/s13019-024-02883-9

**Published:** 2024-06-27

**Authors:** Fan Wei, Chang Yaowen, Wang Wenhui

**Affiliations:** Department of Interventional medicine, The First Clinical Medical College of Lanzhou University, the First Hospital of Lanzhou University, Lanzhou, 730000 Gansu Province China

**Keywords:** Pulmonary artery agenesis, Hemoptysis, Pulmonary hypertension

## Abstract

As the pediatric patient with right pulmonary artery agenesis (PAA) matured, she progressively presented symptoms of pulmonary hypertension and hemoptysis. There is limited clinical literature on this condition, and currently, there is no consensus regarding its diagnosis and treatment. This article presents a case study of a 16-year-old female patient with right pulmonary artery hypoplasia, providing a comprehensive summary and analysis of her developmental progression, pathology, diagnosis, and treatment.

Right PAA is a rare congenital developmental anomaly initially documented by Frantzel in 1868, with a post-mortem incidence of 0.0005% [1]. Over 70% of patients present with associated congenital heart disease, encompassing common malformations such as tetralogy of Fallot, atrial septal defect, coarctation of the aorta, persistent pulmonary trunk, aortic stenosis, transposition of the great arteries, pulmonary atresia, and pulmonary stenosis [2, 3]. The prevalence of right PAA is double that of the left; however left PAA demonstrates stronger association with congenital heart disease [4]. Diagnosis typically occurs during childhood for most patients and common complications include recurrent pulmonary infections, hemoptysis, chest pain and Eisenmenger syndrome. Clinical indicators facilitating early diagnosis comprise features like pulmonary hypertension and congestive heart failure [5]. The mortality rate for individuals with right PAA experiencing pulmonary hemorrhage ranges from 27 to 48% [2, 6], further increasing when accompanied by congenital heart disease.

The 16-year-old female patient presented with intermittent hemoptysis over a three-day period, estimated at approximately 150 ml. The episodes occurred seasonally during winter. She was diagnosed with right PAA thirteen years ago and has exhibited limited exercise tolerance (less than 150 m), along with symptoms of anemia, palpitations, and a preference for squatting. Blood tests revealed the following results: pH: 7.503, pCO₂: 21.80mmHg, sO₂: 90.00%, pO₂: 58.60mmHg, red blood cells: 2.90 × 10¹²/L, hemoglobin:46 g/L; prothrombin time:16.2s; international normalized ratio:1.48; brain natriuretic peptide:533pg/ml. Enhanced chest CT indicated abnormalities in the right pulmonary artery (Fig. 1) and multiple small vessels originating from the neck arteries in the mediastinum and right lung parenchyma. Echocardiography suggested congenital right PAA with patent ductus arteriosus (PDA)(measuring approximately 9 mm in width with shunt velocity of 223 cm/s and pressure gradient of 20mmHg), mild narrowing of the descending aorta leading to secondary pulmonary hypertension (estimated systolic pressure in the pulmonary artery at rest is 83mmHg = brachial systolic pressure—left-to-right shunt pressure difference), main pulmonary trunk diameter measures 31 mm; overall cardiac enlargement is noted particularly on the right side forming a D-shaped configuration; trivial tricuspid regurgitation is observed along with slight dilation of the right coronary artery; minimal pericardial effusion present; both lungs exhibit diffuse interstitial edema. The patient’s WHO functional class was III while her ESC/ERS risk stratification for pulmonary hypertension was intermediate risk level. To control hemoptysis and alleviate pulmonary arterial hypertension selective embolization therapy targeting systemic collateral arteries was performed via Seldinger puncture using a 5 F catheter (YASHIRO) through the right femoral artery. The angiogram showed abnormal communication between bronchial arteries-pulmonary arteries(Fig. 2); visualization also demonstrated anomalous connections between branches such as thyrocervical trunk, internal mammary, and intercostal arteries to spinal cord arteries(Figs. 3 and 4). Embolization using polyvinyl alcohol particles(350–560 μm) and gelatin sponge particles(350–560 μm、1000–1400 μm) was conducted to occlude these systemic collateral vessels, resulting in no residual abnormal systemic circulation after embolization. Tadalafil, a phosphodiesterase 5 inhibitor, and guanylate cyclase stimulator were administered post-embolization to assist in reducing pulmonary arterial pressures. During follow-up at six months, the patient reported no further episodes of hemoptysis, enjoyed normal physical activity(with continuous exertion exceeding 6 h including running without limitation).

**Fig. 1:**
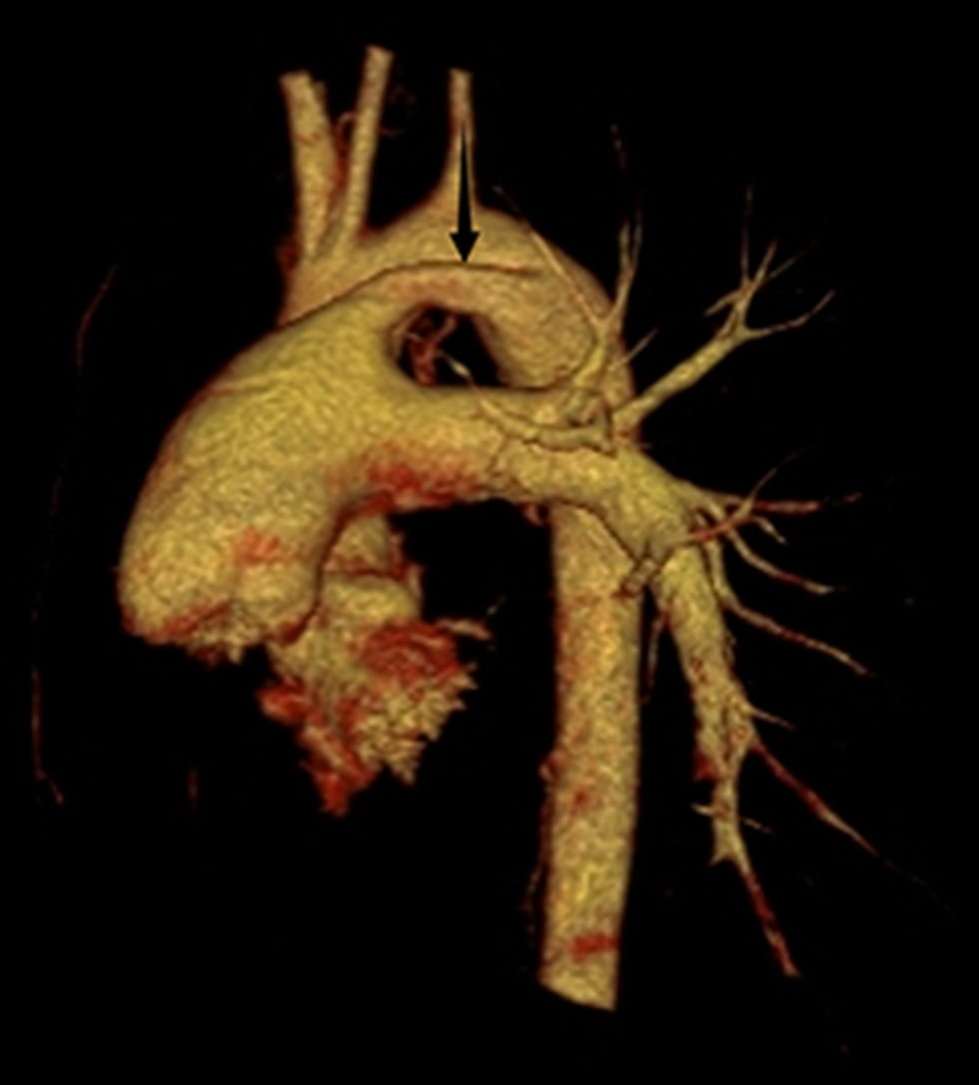
The chest CT scan with three-dimensional reconstruction of a 13-year-old female revealed the absence of the right pulmonary artery (arrow) and patent ductus arteriosus

**Fig. 2:**
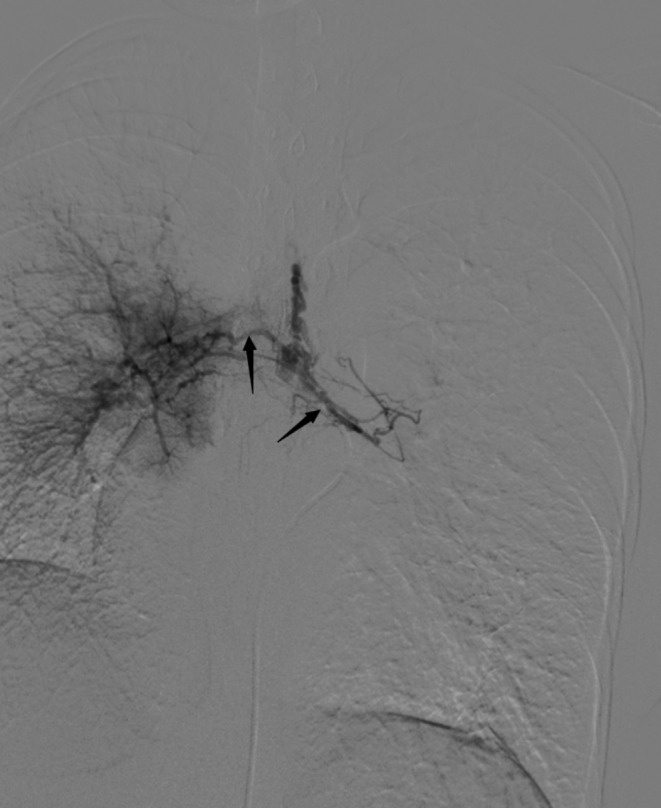
Left (right arrow) and right (left arrow) bronchial arteries open at the thoracic level of T5

**Fig. 3:**
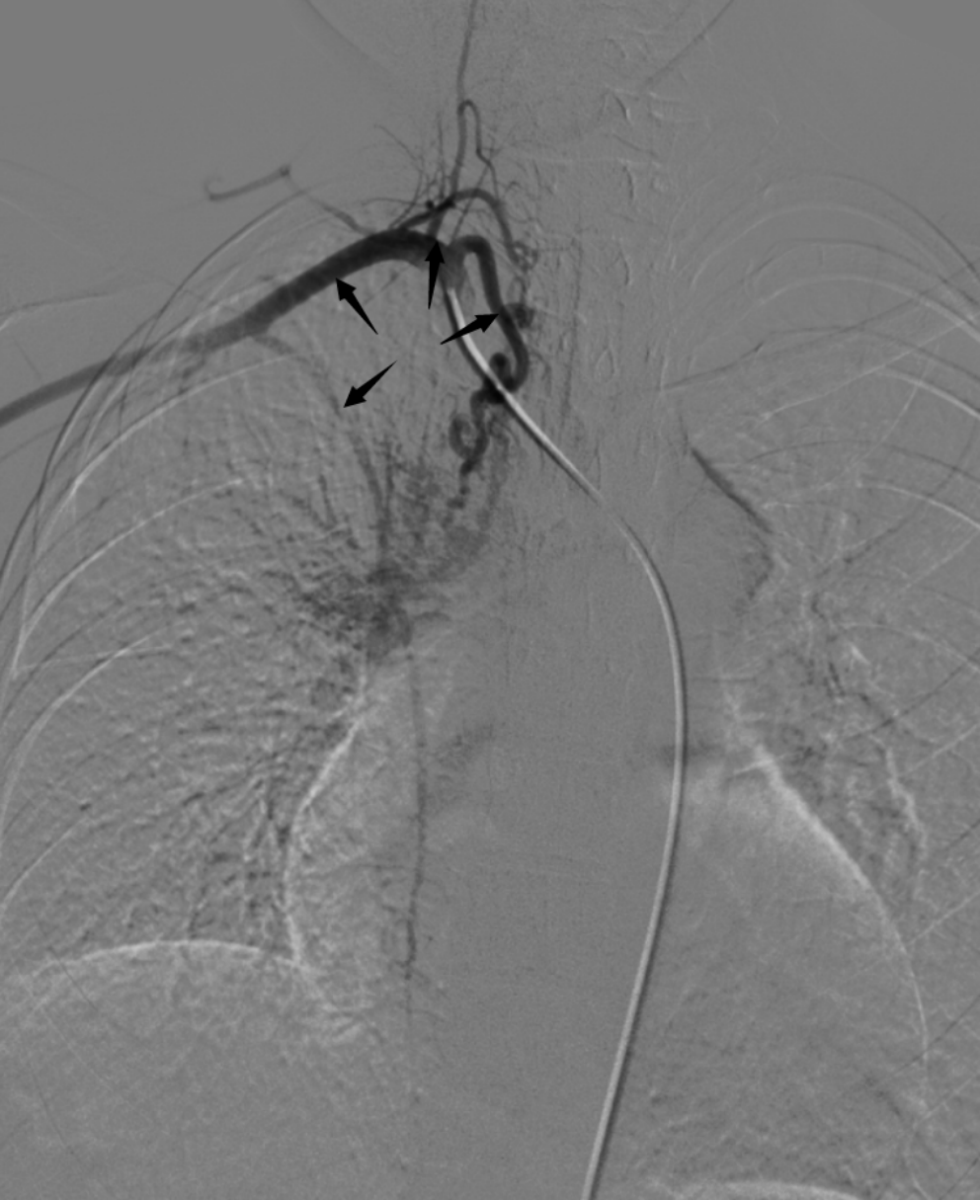
Right costocervical trunk, subclavian artery, thyrocervical trunk, and internal thoracic artery were developed (arrows from left to right)

**Fig. 4:**
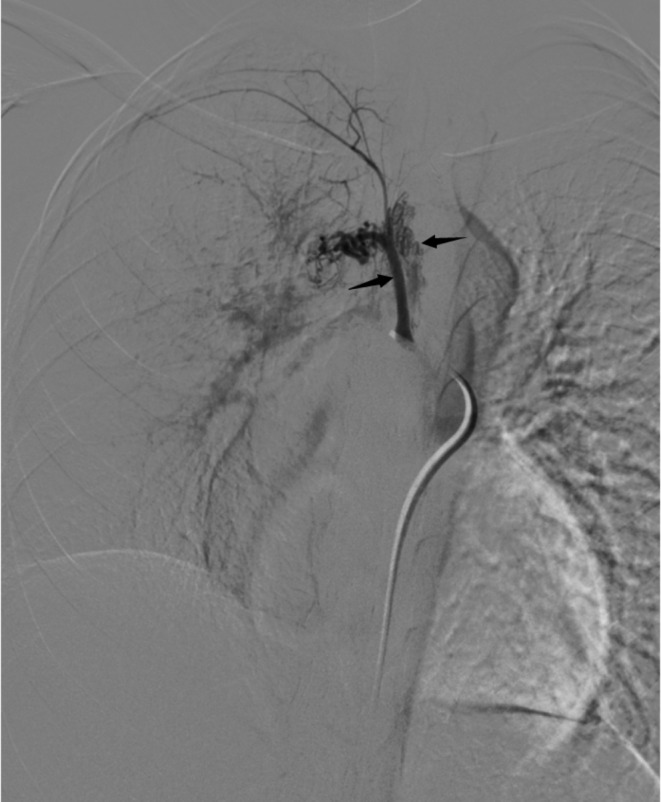
Right intercostal artery (left arrow) and spinal artery (right arrow) were developed

## Development and pathology

During embryonic development, the proximal portion of the bilateral sixth aortic arch undergoes differentiation into the main trunk and left and right branches of the pulmonary artery, while the distal portion on the left transforms into the ductus arteriosus and the right portion regresses [1, 6]. If there is a failure in primitive displacement and rotation of the sixth aortic arch, it results in discontinuity between its proximal portion and the pulmonary artery within the lung. However, originating from the pulmonary bud, the distal portion of the pulmonary artery can maintain a certain degree of branching structure, providing an anatomical basis for abnormal blood supply from bronchial and other systemic arteries. PAA are typically accompanied by varying degrees of underdevelopment in ipsilateral lung tissue, which impacts systemic circulation arteries’ origin and distribution [7]. Abnormal systemic circulation arteries may originate from bronchial, intercostal, intrathoracic, thyrocervical trunk, subclavian, or hypogastric arteries; fewer cases have been reported involving blood supply from coronary arteries. In children with unilateral PAA where all cardiac output flows to one lung side due to excessive blood flow increasing shear stress on endothelial cells leading to long-term decline in vascular bed elasticity as well as excessive sensitivity to vasoconstrictive substances [8]. Additionally reduced relative size of affected side’s pulmonary vascular bed along with continuous opening of systemic circulation arteries leads to increased risk for adult-onset pulmonary hypertension that tends to stabilize as open arterial numbers stabilize during adulthood [8].

## Diagnosis

Children with PAA may develop recurrent pulmonary infections (37%), reduced exercise tolerance (40%), pulmonary hypertension (44%), and hemoptysis (20%) as they age, or remain asymptomatic until adulthood [3, 9, 10]. Physical examination may reveal chest asymmetry, abnormal breath sounds on the affected side, and a generally normal electrocardiogram. When a chest X-ray shows increased lung transparency and hypoplasia of the lung (contralateral displacement of the trachea, fewer pulmonary vascular segments), along with fine linear opacities in the peripheral lung, suspicion of PAA is high; however, it needs to be differentiated from diseases causing diffuse changes in one lung field such as bronchiectasis, hypoplastic lung, scimitar syndrome, Swyer-James syndrome, mediastinal tumor, fibrotic mediastinitis, and pulmonary venous occlusive disease [11, 12]. CT three-dimensional reconstruction is an important preoperative diagnostic method; angiography is reserved for individuals requiring surgical intervention due to its invasiveness [2]. Common methods for measuring pulmonary artery pressure include tricuspid regurgitation velocity method, pulmonary valve regurgitation method, and intracardiac shunt method. Right ventricular enlargement and thickening as well as widening of the right ventricular outflow tract are suggestive but lack diagnostic significance for patients with unilateral absence of a pathway in the pulmonary artery [13]. In this case study patient’s right subclavian artery systolic pressure was used along with catheter left-to-right shunt pressure difference for estimation.

## Treatment of disease

Refined sentence: The literature on PAA leading to pulmonary hypertension is primarily based on case studies and adult clinical staging. Early diagnosis and intervention are essential principles. Treatment aims to reduce risk stratification (low-risk) and ensure unrestricted normal activities. Prognosis depends largely on the type of cardiovascular disease associated with pulmonary hypertension and the severity of the condition [14]. In children with right PAA hypoplasia, the ductus arteriosus maintains a balance between systemic and pulmonary circulation blood flow, ensuring cardiac function. While ligation is considered the optimal treatment for PDA patients, it may further restrict pulmonary circulation, disrupt equilibrium, and impede oxygen exchange [15]. This limitation has been a primary reason for not performing ductus arteriosus ligation thus far; currently, monitoring cardiovascular hemodynamics is deemed most beneficial [16, 17]. A retrospective study suggests that early ductus arteriosus ligation is linked to severe bronchial artery hypoplasia; children with right pulmonary artery hypoplasia are likely to exhibit more severe symptoms [18].The insufficient blood flow in the right lung leads to a decreased delivery of phagocytes to the inflammatory site, reduced carbon dioxide exchange rate, and secondary bronchial constriction and mucus retention, resulting in recurrent pulmonary infections. While systemic circulation improvement enhances pulmonary perfusion to some extent, it also elevates the risk of pulmonary hypertension and hemoptysis [7]. Hemoptysis occurs spontaneously over several years and often follows a seasonal pattern, worsening with age [19]. The most optimal treatment for children with PAA involves utilizing autologous pericardium or artificial materials for pulmonary artery vascular reconstruction. This approach can promote lung development and alleviate vascular system damage as long as the relative integrity of the pulmonary arterial tree is maintained. However, challenges arise due to mismatch between implant size and the child’s developing body, necessitating staged procedures. Any lung surgery for a child with such anomalies will be complicated by PDA and open systemic arteries. In cases where surgical removal of infected sites fails to control recurrent pulmonary infections and hemoptysis, unilateral lung resection becomes the last resort option. Unfortunately, mortality rates are extremely high after unilateral lung resection among patients with accompanying anemia, pulmonary hypertension, and acute respiratory failure due to these anomalies. Selective systemic circulation arterial embolization proves effective in controlling coughing fits along with managing both pulmonary hypertension and infection [15], offering good hemostatic effects while minimizing trauma; this procedure can be repeated if necessary. It is crucial to ensure complete embolization of responsible vessels in order to avoid missed embolizations that could lead to higher recurrence rates and mortality [20, 21].Following selective embolization of the systemic arteries, there was a reduction in blood flow in the right pulmonary circulation and a decrease in pulmonary arterial pressure, with no significant impact on oxygen exchange or lung lobe development (16 years old). For abnormal open systemic circulation arteries (arterial fistula), larger diameter permanent solid embolic agents are preferred due to their irregular surfaces that are more prone to clotting and can prevent occlusion of aberrant small vessels. In cases of suspected spinal or coronary arteries, non-permanent embolic agents or no embolization is recommended. Embolization should be followed by targeted drug therapy as per the 2022 ESC/ERS Guidelines for the diagnosis and treatment of pulmonary hypertension and the 2021 Chinese Guidelines for the Diagnosis and Treatment of Pulmonary Hypertension [22]. These guidelines recommend single drug therapy for patients with pulmonary hypertension; low-risk group patients should receive endothelin receptor antagonists (such as Bosentan), while high-risk group patients should be given prostacyclin analogues and prostacyclin receptor agonists (such as Epoprostenol). The patient in this case belongs to a moderate-risk group and was administered Phosphodiesterase 5 inhibitors and guanylate cyclase stimulators (such as Tadalafil). If clinical symptoms do not improve after single drug therapy, early combination targeted drugs can be considered, especially for WHO functional grade IV patients who may benefit from an initial combined treatment strategy. Supportive treatments such as diuretics and oxygen therapy can also be provided, along with long-term use of vasodilators which have been shown to improve survival rates [23, 24].

## Conclusion

In conclusion, for pediatric patients with PAA and PDA, selective embolization of systemic circulation arteries can alleviate pulmonary hypertension and represents a viable therapeutic option for those ineligible for revascularization. Continuous monitoring of cardiovascular hemodynamic changes is advised, while early ductus arteriosus ligation is not recommended.

## Data Availability

No datasets were generated or analysed during the current study.
